# Utilization of Murine Laparoscopy for Continuous *In-Vivo* Assessment of the Liver in Multiple Disease Models

**DOI:** 10.1371/journal.pone.0004776

**Published:** 2009-03-10

**Authors:** Yami Shapira, Meirav Katz, Muhammad Ali, Michael Kaplan, Eli Brazowski, Zamir Halpern, Eran Elinav

**Affiliations:** Institute for Gastroenterology and Liver Disease, Tel Aviv Sourasky Medical Center, Tel Aviv, Israel; Karolinska Institutet, Sweden

## Abstract

**Background:**

Current strategies for follow up of murine models of liver disease are flawed by inability to continuously monitor disease progression in the tissue level, and necessitate sacrifice of animals for tissue sampling.

**Aims:**

In this study we aimed at developing a safe repetitive tool for sampling livers in vivo, by utilization of a miniaturized endoscopy system for laparoscopic liver biopsies and for injection of tumor cells into livers.

**Results:**

We report the development of a protocol for murine laparoscopy that allows repeated visualization of murine intra-abdominal organs. The system enables safe and repeated liver biopsies in mice and rats, yielding adequate tissue for histological staining and RNA extraction. In addition, injection of tumor cells into livers facilitates under-vision implantation of hepatic tumors in liver, followed by visualization of tumor growth.

**Conclusions:**

Murine laparoscopy may be employed as a novel imaging modality for continuous assessment and manipulation of chronic liver disease models.

## Introduction

Murine models are a major and frequently employed tool for investigating mechanisms of liver disease. Multiple such models are widely used for investigation of hepatic inflammation, steatosis, fibrosis and tumorogenesis. The nearly universal course of such experiments is sacrifice of animals at the end of the experiment and assessment of the desired end points using histological and molecular measures. This structure has several pitfalls. High mortality rates in many models of acute and chronic liver disease necessitate large numbers of mice in each experimental group. Inter-animal variability makes the numbers needed to achieve meaningful and statistically significant results even greater. Consecutive follow-up of disease development and progression in individual mice is impossible, resulting in utilization of surrogate parameters of disease activity such as weight loss, loss of activity and mortality. While clinically important, such parameters are difficult to predict and mechanistically assess relevant pathological and cellular processes.

In addition, in primary and metastatic liver tumor models, implantation of tumor cells into original target organs, especially deep within the abdominal cavity, can be technically challenging, often necessitating tumor implantation in alternative, remote locations, most often subcutaneously[1]. Such approaches disregard the effect of the local milieu on tumor environment, thus limiting the correlation between the model and naturally-occurring tumors[2], [3]. Liver tumors are fine examples of such limitations, as intra-hepatic implantation of tumors in small animals is achievable either by penetrative operational techniques (associated with high mortality rates), or by intra-splenic injection of tumor cells and hepatic metastasis which is associated with unpredictable numbers of tumor cells arriving to the liver parenchyma[4].

In this article, we describe a novel approach to combat these technical limitations, using an easy to employ, safe and reproducible murine laparoscopy technique. We suggest that application of this approach may provide the investigator with the tools to assess and manipulate disease progression in-vivo in individual mice, allowing for a more comprehensive understanding of mechanisms of disease progression.

## Methods

### Animals

Animals had unrestricted access to food and water, were housed in temperature and humidity-controlled rooms, and were kept on a 12-hour light/dark cycle. All animal studies were approved by the Tel Aviv Sourasky Medical center ethical committee for animal studies and conformed to the highest international standards of humane care of animals in biomedical research.

### Experimental procedures

8–10 week-old female C57BL/6 and Balb C mice and Wister rats were obtained from Harlan biotech (Rehovot, Israel). Methionine Choline Deficient diet (MCD Diet, MP Biomedicals, Ohio, USA) was administered to C57BL/6 mice ad libitum for 8 weeks to induce NASH as described elsewhere[5]. Thioacetamide liver fibrosis was induced in C57BL/6 mice by 8 weeks of thrice-weekly intraperitoneal injection of 200 mg/kg Thioacetamide (Sigma co., Rehovot, Israel)[6]. Green Fluorescent protein (GFP) or Luciferase stably- transfected murine hepatic tumor cells (BNL1ME) were kindly provided by Prof. Eithan Galun (Hadassah-Hebrew University Medical Center).

### Murine laparoscopic system

We employed a high resolution mouse video endoscopic system (“Coloview system”) previously described for murine endoscopic procedures[7], which consists of a miniature endoscope (scope 1.9 mm outer diameter), a xenon light source, a triple chip camera, and an air pump to achieve regulated inflation of the mouse peritoneal cavity (Karl Storz, Tuttlingen, Germany). The endoscopic procedure was viewed on a color monitor and digitally recorded on tape. Biopsies were performed using a flexible biopsy forceps with a diameter of 3 Fr. Intrahepatic injections were accomplished using a 2 French catheter with a 25 G needle fitted at its end, inserted through the working channel of the scope. Biopsies were immediately placed in formaldehyde or liquid nitrogen.

### Murine laparoscopic procedure

100 mcg/g Ampicillin and 5 mcg/g Gentamycin were subcutaneously administered to all mice peri-interventially, for a total period of 2 days. Mice were anesthetized using Ketamine/Xylazine. Aseptic technique was strictly followed. Skin incision was made using scissors and scalpel, forming a minimal incision two millimeters to the left (left lateral laparoscopy) or to the right (right lateral laparoscopy) of the midline, at the epigastric region. The incision was 1 mm in size, large enough to accommodate the scope (approximately 1.5 mm). Air was insufflated through the laparoscope into the peritoneal cavity to achieve adequate vision of the abdominal cavity, and pneumoperitoneum was maintained by the tight incision. By the end of the procedure, both operative peritoneal and skin incisions were sutured using a single 3/0 silk suture, respectively.

### Histological examination

Hepatic biopsies and sections from excised liver were stained with Hematoxylin and Eosin (all sections) and Sirius Red stain (in fibrosis experiments). All sections were reviewed by an expert blinded pathologist.

### RNA extraction

Total cellular RNA from liver tissue was extracted with EZ-RNA total RNA isolation kit (Biological Industries, Beit Haemek, Israel) and transcribed into cDNA, using the Reverse Transcription System (Sigma Aldrich, Rehovot, Israel). mRNA for albumin and GADPH were obtained after 35 cycles of amplification (annealing temperature of 56–62°C) using the following primers:

Albumin: Forward- CCTGATTGCCTTTTCCCAGTATCTCCAG, Reverse- CCAATGCTTTCTCCTTCACACCATCA, GAPDH: Forward- AACTTTGGCATTGTGGAAGG, Reverse- CACATTGGGGGTAGGAACAC.

Amplification products were visualized by ethidium bromide after agarose electrophoresis and semiquantified by visually after normalization against the GADPH internal control.

### In vivo imaging

To follow luciferase-positive tumor implantation and growth, the whole body cooled CCD camera system was used (IVIS® 100 Series Imaging System, Xenogen, Alameda CA), as was previously described[8]. For in-vivo visualization of GFP-positive tumors, the Axioimager-M (Carl Zeiss, Oberkochen, Germany) fluorescent microscopy system was used.

## Results

### Safety of procedures

Murine laparoscopy was performed in 150 mice. Laparoscopic procedures were successfully performed in naïve mice as well as in mice with TAA-fibrosis, MCD-deficient diet NASH, and liver tumor models. After a short learning curve procedures could be accomplished safely and reproducibly in all models. No perioperative mortality was noted in first-time biopsies, while repeat procedures (performed in two week intervals) were associated with a total 10% mortality rate, attributed to abdominal adhesions from previous procedures, resulting in increase procedure time and associated bleeding and anesthetic complications. No episodes of infection were noticed in either first time or repeat procedures. We now routinely perform 5 and more weekly repetitive laparoscopies in diseased animals.

### Visualization of abdominal organs by murine laparoscopy

**Figure 1:**
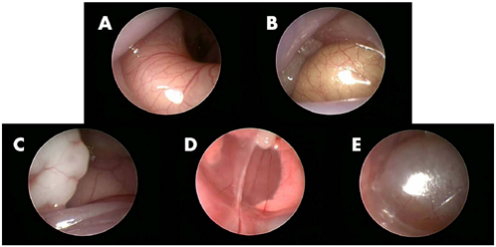
Right lateral laparoscopic imaging of mouse intra-abdominal organs. A – Large intestine, B – Small intestine, C – Ovary, D – heart and lungs viewed through diaphragm, E – urinary bladder.

**Figure 2:**
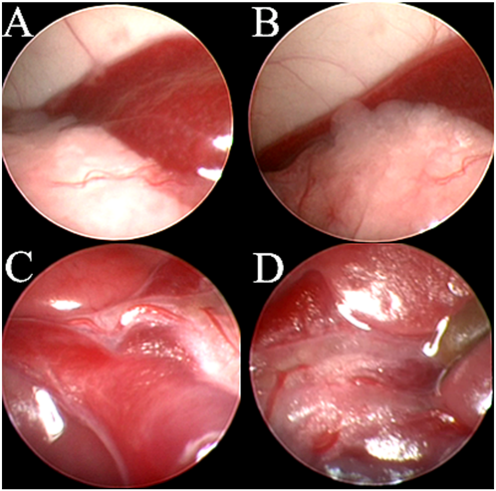
Left lateral laparoscopic imaging of mouse intra-abdominal organs. A – Spleen, B – Pancreas overhanging the spleen, C – Bifurcation of the portal vein, D – Cystic duct and gallbladder.

**Figure 3:**
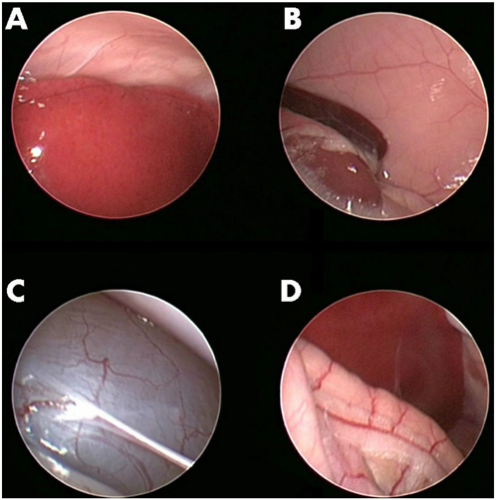
Laparoscopic imaging of rat intra-abdominal organs. A- Liver, B-spleen and upper pole of left kidney, C- urinary bladder, D- hepatic flexure of colon.

As seen in **figure 1** (right lateral laparoscopy), **figure 2** (left lateral laparoscopy) and **video S1**, high quality visualization of internal abdominal organs could be easily achieved. **Figure 3** depicts views obtained by right lateral laparoscopy of Wister rats that yielded similar results. Visualized organs included the small and large intestine, liver including the porta hepatis, gall bladder and associated major vessels, spleen, pancreas, diaphragm (with the beating heart and lungs visualized above it), urinary bladder, and ovaries.

### Laparoscopic macroscopic appearance of the liver in health, NASH, and fibrosis

**Figure 4:**
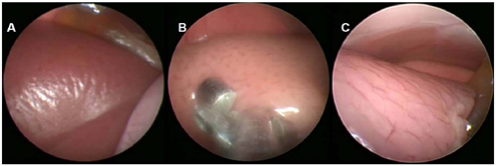
Laparoscopic imaging of healthy and diseased livers. A – Healthy liver, B – MCD Diet-induced NASH (with biopsy forceps in frame), C – TAA-induced liver fibrosis.

As is depicted in **figure 3** and **video S2**, high resolution imaging of the liver in health and in various disease states could be achieved using murine laparoscopy. While healthy livers appear violet in color and smooth in texture (**figure 4A**), severe steatosis results in white discoloration (**figure 4B**), while fibrosis results in a gray discoloration of the liver and intense granulation and irregularities of its surface (**figure 4C**). In tumor models, subscapular masses could be easily detected within days of implantation (see below).

### Laparoscopic biopsy-taking and histological examination

**Figure 5:**
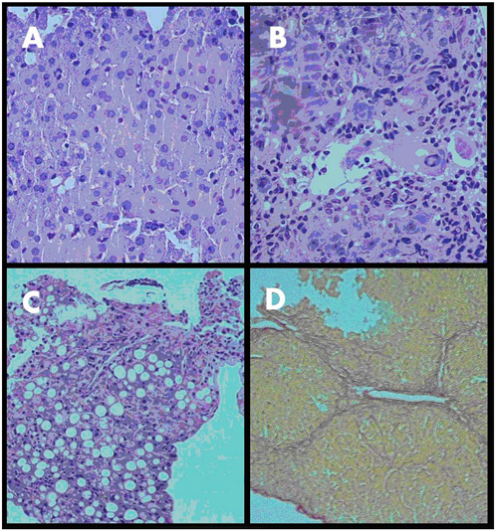
Histological sections from biopsies taken during laparoscopy. A – normal liver (×100, H&E), B – TAA induced fibrosis (×100, H&E), C - MCD Diet induced NASH (×40, H&E), D - TAA induced fibrosis (×40, Serius Red).

Liver biopsies were taken by a miniaturized biopsy forceps that was inserted through the endoscope working channel. Biopsy taking necessitated the presence of two examiners, one guiding the endoscope to the desired liver segment, the other controlling the opening and closure of the biopsy forceps. Multiple biopsies could be undertaken, each lasting approximately 30 seconds. Mild, self-contained bleeding validated that the liver tissue was indeed biopsied (**video S3**). No mortality was associated with the biopsy. Biopsy size was 3–7 mm in average. Time requirements for a single laparoscopy for liver examination are around 45 seconds per mouse. A biopsy prolongs the procedure by up to one extra minute. **Figure 5** depicts the histological staining of representative biopsy specimens from normal liver (**figure 5A**, H&E stain), MCD-induced non-alcoholic liver (**figure 5B**) showing an area of intense lymphocytic exudate, **figure 4C** showing an area of severe steatosis, (H&E stain) and a liver with TAA-induced fibrosis (**figure 5D**, Sirius red stain). In all biopsy specimens, full correlation was observed with corresponding histological slides of livers from the same mice (data not shown).

### Laparoscopic collection of hepatic mRNA

**Figure 6:**
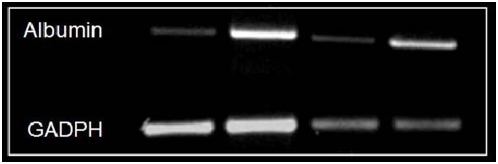
rtPCR from hepatic mRNA collected by laparoscopic biopsy. In all four biopsied mice, albumin and GADPH mRNA expression could be clearly detected.

Biopsy specimens were also used for extraction of total liver mRNA. In average, a single biopsy produced approximately 2 micrograms mRNA. This quantity of mRNA enabled the assessment of multiple liver-specific or liver-non-specific gene transcription products (such as albumin and GADPH, depicted in **figure 6**).

### Laparoscopic local injection of tumor cells into the liver

**Figure 7:**
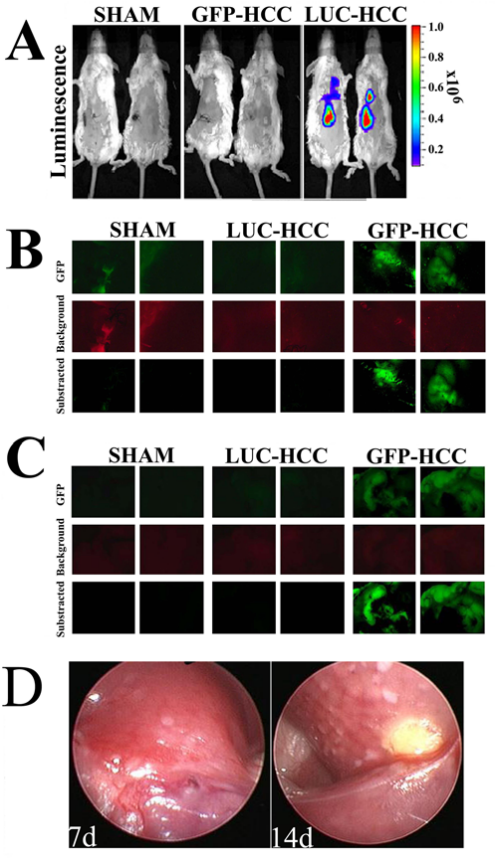
Liver tumors implanted during murine laparoscopy. A. In-vivo imaging (IVIS 100) of hepatic luciferase signal in sham operated, GFP-HCC implanted or luciferase-HCC implanted mice. B. In-vivo imaging (ZIESS Axioimager-M) of hepatic GFP fluorescence in sham operated, GFP-HCC implanted or luciferase-HCC implanted mice. C. Repeated GFP imaging (ZIESS Axioimager-M) in excised livers of sham operated, GFP-HCC implanted or luciferase-HCC implanted mice. D. Laparoscopic appearance of liver-infiltrating GFP-HCC tumors 7 & 14 days following hepatic tumor cell implantation.

One of the most attractive usages of murine laparoscopy is the accurate guided implantation of tumor cells within target organs. To demonstrate such ability, 5×106 GFP or Fluorscin-positive BNL1ME hepatocellular carcinoma cells were suspended in 200 microliter of HBSS and directly injected into the right hepatic lobe through the murine laparoscope working channel (**video S4**). Using the IVIS in-vivo imaging system for luminescence detection, the fluorescin-positive tumor could be visualized within the liver as soon as 3 days after implantation (data not shown), and was brightly-positive in all luciferase-tumor harboring mice, but in non of the sham-operated or GFP-positive mice, 10 days after tumor implantation (**figure 7A**). Inversely, using the Carl Zeiss Axioimager-M fluorescent microscopy system for in-vivo detection of GFP signals, in the two mice in which a GFP-positive tumor was implanted, intense fluorescent signal could be detected, while no signal was omitted from either sham or fluorescin-positive tumors (**figure 7B**). Liver excision from all mice confirmed that the GFP signal, detected in-vivo, indeed originated from the hepatic tumors (**figure 7C**). Tumors could be repetitively examined and biopsied using routine weekly laparoscopy (**figure 7d**). In our hands, tumor implantation by left lateral laparoscopy followed by repeated visualization of tumor growth by right lateral thoracotomy yield the most reproducible results.

## Discussion

We describe herein a novel approach for monitoring the initiation and progression of liver disease in murine and other small animal models, using a commercially available miniendoscopy system and a simple and safe laparoscopic technique. We demonstrate that murine laparoscopy enables safe and repeated visualization of a diverse variety of intra-abdominal organs. Focusing on the liver, we show that our approach enables early detection and follow up of macroscopic morphological changes in several models of hepatic pathology and that laparoscopic liver biopsies can reliably assess the pathological stage of disease, and produce mRNA for molecular assessment of hepatic gene expression. Moreover, we show that laparoscopy-guided local injection of tumor cells into the liver results in in-situ development of liver tumors. Most important, our approach enables repeated visualization and intervention within the same mouse, such that inter-animal variability can be avoided and detection of temporal changes can be optimized.

Chronic liver disease and cirrhosis represent a common worldwide health problem, affecting large and growing numbers of the world population, causing a major burden on society's health expenditures. Common hepatic disorders, such as viral hepatitis and non alcoholic steatohepatitis, are major risk factors for the development of cirrhosis and hepatocellular carcinoma[9]. It has been well described in humans with liver disease that use of laparoscopy as part of the evaluation of liver disease increases the sensitivity of disease assessment[10], [11].

Both MCD diet-induced and Thioacetamide-induced murine liver disease models, employed in our study, are widely used for investigation of human NASH and chronic liver fibrosis, respectively. Although a MCD dietary model does not replicate in full measure the pathogenesis of NASH in humans, it represents one of the best-established methods to study the evolution of inflammation, oxidant stress, and fibrotic changes associated with NASH[12]. TAA induced liver fibrosis is considered a valid model, simulating human cirrhotic histology and established portal hypertension[13].

We believe that laparoscopic techniques in experimental liver disease models may be particularly useful in states in which mechanisms of disease progression are hard to elucidate in state of high ‘background noise’ secondary to variations of disease severity between individuals. Non alcoholic steatohepatitis is one fine example, in which different stages of disease can be detected within the same liver[14], and even more so between different animals. Understanding of progression of simple steatosis to steatohepatitis and chronic liver disease may be facilitated if livers of the same animals be serially sampled for histological and molecular markers, rather than relying on post mortem samples of sibling animals that are frequently associated with significant inter-animal variability.

Another advantage in such cases is the possibility of sampling at early stages of disease, when pathological changes are mild to non-existent, while continuing the follow up for subsequent disease progression. In addition, tumor injection into predefined target organs enables the incorporation of the important and often disregarded aspect of the microenvironment local effects on tumor growth and the tumor-environment crosstalk.

Limitations of the laparoscopic method include a certain (albeit very low) rates of mortality of the procedure, mainly during the learning curve phase of the procedure, and possible effects of antibiotics and the sedation and trauma of the procedure on the examined animal. Nevertheless, incorporation of murine laparoscopy into the scope of in-vivo examinations enables the much desired sensitive and repeatable online assessment of live small animals, while dramatically reducing the need for large animal groups for each experiment. In the near future, integration between the laparoscopy approach and other in vivo imaging modalities[15], [16] may further enhance our ability for real-time assessment of disease processes. In conclusion, murine laparoscopy is a novel imaging modality that enables safe and effective continuous assessment of chronic inflammatory, metabolic and neoplastic liver disease models in mice.

## Supporting Information

Video S1 Laproscopic examination of abdominal organs
(2.12 MB MOV)

Video S2 Laparoscopic macroscopic appearance of the liver in health, NASH, and fibrosis
(3.30 MB MOV)

Video S3 Laparoscopic liver biopsy taking
(0.84 MB MOV)

Video S4 Laparoscopic local injection of tumor cells into the liver
(4.37 MB MOV)
